# *OsRACK1* Is Involved in Abscisic Acid- and H_2_O_2_-Mediated Signaling to Regulate Seed Germination in Rice (*Oryza sativa*, L.)

**DOI:** 10.1371/journal.pone.0097120

**Published:** 2014-05-27

**Authors:** Dongping Zhang, Li Chen, Dahong Li, Bing Lv, Yun Chen, Jingui Chen, XuejiaoYan, Jiansheng Liang

**Affiliations:** 1 Department of Biotechnology, College of Bioscience and Biotechnology, Yangzhou University, Jiangsu, China; 2 Department of Biological Engineering, Huanghuai University, Zhumadian City, Henan Province, China; 3 Biosciences Division, Oak Ridge National Laboratory, Oak Ridge, Tennessee, United States of America; Institute of Botany, Chinese Academy of Sciences, China

## Abstract

The receptor for activated C kinase 1 (RACK1) is one member of the most important WD repeat–containing family of proteins found in all eukaryotes and is involved in multiple signaling pathways. However, compared with the progress in the area of mammalian RACK1, our understanding of the functions and molecular mechanisms of RACK1 in the regulation of plant growth and development is still in its infancy. In the present study, we investigated the roles of rice RACK1A gene (*OsRACK1A*) in controlling seed germination and its molecular mechanisms by generating a series of transgenic rice lines, of which *OsRACK1A* was either over-expressed or under-expressed. Our results showed that *OsRACK1A* positively regulated seed germination and negatively regulated the responses of seed germination to both exogenous ABA and H_2_O_2_. Inhibition of ABA biosynthesis had no enhancing effect on germination, whereas inhibition of ABA catabolism significantly suppressed germination. ABA inhibition on seed germination was almost fully recovered by exogenous H_2_O_2_ treatment. Quantitative analyses showed that endogenous ABA levels were significantly higher and H_2_O_2_ levels significantly lower in *OsRACK1A*-down regulated transgenic lines as compared with those in wildtype or *OsRACK1A*-up regulated lines. Quantitative real-time PCR analyses showed that the transcript levels of *OsRbohs* and amylase genes, *RAmy1A* and *RAmy3D*, were significantly lower in *OsRACK1A*-down regulated transgenic lines. It is concluded that *OsRACK1A* positively regulates seed germination by controlling endogenous levels of ABA and H_2_O_2_ and their interaction.

## Introduction

Seed germination is a complex processes that is under combinatorial control by endogenous and environmental cues. Because seed germination is a critical stage in the plant life cycle and is the first step towards successful plant establishment, it is of importance to unravel the physiological and molecular mechanisms underlying seed germination control in order to genetically manipulate it. A large number of studies have been conducted to explore seed germination mechanisms over the past several decades [1]–[4]. While there is much information with respect to changes in cellular events and molecular processes during germination, neither the key event(s) nor the regulatory network has been identified that results in its completion [5]. Recently, transcriptomic, proteomic and metabolomic analyses have proved that transcriptional, translational and post-translational modification in *Arabidopsis* seeds are more complex and dynamic than previously thought [6]–[9],[3],[10],[11]. Bassel et al.[7], using publicly available gene expression data from *Arabidopsis thaliana*, generated a condition-dependent network model of global transcriptional interactions associated with seed dormancy or germination and identified 1,583 transcripts were associated with germination. He et al.[3] analyzed the protein profiling in the germinating rice seeds through 1-DE via LC MS/MS proteomic shotgun strategy and identified that a total of 673 proteins were involved in seed germination. These proteins could be divided into 14 functional groups and the largest group was metabolism-related. Others included many regulatory proteins which control cellular redox homeostasis and gene expression of the germinating seeds.

In addition to the intensive changes both in gene expression and in metabolic activities during seed germination, the levels of plant hormones and many signaling molecules, such as reactive oxygen species (ROS), NO, etc, also changed drastically during seed development and seed imbibition in response to developmental and environmental cues [2], [12]–[14]. Abscisic acid (ABA) and gibberellins (GAs) were considered as two major hormones controlling seed dormancy and germination [15],[16]. ABA promotes the induction and maintenance of seed dormancy whereas GA is required for the initiation and completion of germination. It is thought that germination is regulated by the antagonistic effects between ABA and GA. However, the regulation of ABA and GA on seed germination was much more complex than it was thought. For example, Okamoto et al. [17] found that, although application of exogenous ABA inhibited germination, the effects of exogenous ABA on ABA-mediated gene transcription differ from those of endogenous ABA. The effects of exogenous ABA were prominent in the expression of several ABA-related genes at a later stage of imbibition, whereas the endogenous ABA affected the expression of critical components, e.g. ABA signaling, photosynthesis, physiological and metabolic genes including a GA biosynthesis enzyme.

Although ROS are long considered hazardous molecules, their functions as cell signaling compounds are now well established and widely studied in plants[4],[18]. Great fluctuations of ROS levels occur continuously during seed development and germination. Growing evidence have shown that ROS may function as messengers or transmitters of environmental cues during seed germination and positively control seed germination [4],[19]–[24]. Little is currently known, however, about ROS biochemistry or their functions or the signaling pathways during these processes.

The receptor for activated C kinase 1 (RACK1) is one member of the most important tryptophan, aspartic acid repeat (WD repeat)–containing family of proteins found in all eukaryotes [25]–[27]. As a scaffolding protein, RACK1 is involved in multiple signaling pathways [27]–[30]. The first plant *RACK1* was originally identified as an auxin inducible gene, *arcA*, in tobacco BY-2 suspension cells in a differential screen for genes involved in auxin-mediated cell division [31],[32], and thereafter, RACK1 gene was isolated from a range of plant species, including *Arabidopsis thaliana*, *Oryza sativa*, and found to be expressed ubiquitously in different tissues and organs, including leaf, stem, root, and flower, etc., which implies that RACK1 may play important roles in plant growth and development [28],[33],[34]. The *Arabidopsis* genome contains three RACK1 orthologues, At1g18080, At1g48630, and At3g18130, designated as *RACK1A*, *RACK1B*, and *RACK1C*, respectively. These three *Arabidopsis* proteins are approximately 65% identical and 78% similar to mammalian RACK1 [28]. Chen et al. [28] provided direct genetic evidence of the function of RACK1 in plant responses to several plant hormones using the loss-of-function mutants of *RACK1* in *Arabidopsis*. They found that *rack1a* mutants displayed altered sensitivities to several plant hormones, including hyposensitivity to gibberellic acid and brassinosteroid in seed germination, hyposensitivity to auxin in adventitious and lateral root formation, and hypersensitivity to ABA in seed germination and early seedling development [28]. The ABA-responsive marker genes, *RD29B* and *RAB18*, were up-regulated in *rack1a* mutants and the expression of all three *RACK1* genes themselves was down-regulated by ABA [35]. Islas-Flores et al., using an RNAi approach to suppress the RACK1 gene expression in *P. vulgaris* (*PvRACK1*), and found that mRNA accumulation of *PvRACK1* in roots was induced by auxins, ABA, cytokinin, and gibberellic acid [36],[37]. Our studies on the functions of *Arabidopsis* and rice RACK1 in the responses to drought stress showed that, when RACK1 gene expression was suppressed, leaf ABA level significantly increased, as a result, the tolerance of seedlings to soil drying increased [38]. Growing evidence has shown that RACK1 is critical regulators of plant development and loss-of-function mutations in RACK1A confer defects in multiple developmental processes including seed germination, leaf production, and flowering [28]. The BLASTP search using *Arabidopsis* RACK1A protein (NCBI accession number: NP_173248) as a template revealed that, in rice genome, there are two *RACK1* homologous genes, *OsRACK1A* and *OsRACK1B*, which are approximately 80% similar to *Arabidopsis* RACK1 proteins at the amino acid level. Nevertheless, at present, we know little about whether RACK1 is involved in and how it regulates seed germination, especially in rice. Gene expression profile analysis indicated that *OsRACK1A* gene was highly expressed whereas *OsRACK1B* was lowly expressed in rice seeds (Figure S1). In this experiment, we firstly generated a series of transgenic rice lines, of which *OsRACK1A* gene was either over-expressed or under-expressed, and explored the roles of *OsRACK1* in regulating seed germination. Analysis of these transgenic lines revealed that *OsRACK1A* positively regulates seed germination through enhancing ABA catabolism and promoting H_2_O_2_ production.

## Materials and Methods

### Plant Materials

Rice (*Oryza sativa* L. cv. Nipponbare) was used as the wildtype (non-transgenic line, NTL) and in the generation of all transgenic plants. All transgenic rice lines were generated and kept in our laboratory. *OsRACK1A* over-expression transgenic lines, OeTL3-8 and OeTL4-9, anti-sense transgenic line, AsTL7-6 and RNA-interfered transgenic lines, RiTL3-1, RiTL4-2 and RiTL7-3 were used as experimental materials in this study [38].

### Plant Growth Conditions, Seed Germination Assay and Stress Treatments

Seeds collected from the rice plants of wildtype and the 6^th^ generation of transgenic lines were used in this experiment. Fully-filled and uniformed rice seeds were washed with 70% (v/v) ethanol for 30 seconds, and washed three times with sterile water. Sterilized seeds were subsequently sown on sterile filter papers in the petri dishes (size:150 mm diameter) which contained different concentrations of ABA (0∼40 µM), NaCl (0∼200 mM), Glucose (0∼3%) (w/v), and 20 µM fluridone, 100 µM diniconazole, or 20 mM H_2_O_2_ (Sigma). Stock solution of ABA (mixed isomers, Sigma), fluridone (Sigma) and diniconazole (Sigma) were dissolved in ethanol. Control petri dishes contained equal amount of sterilized water. Seeds were germinated in a growth chamber with 12 h light and 12 h dark at 28°C. Germination (based on radicals >2 mm) was recorded at the indicated time points. For each germination test, fifty seeds per genotype were used, and three experimental replications were performed. The average germination percentage ± SE (standard error) of triplicate experiments was calculated. Seeds imbibed for different time in the presence of water or ABA were collected and stored at −80°C for ABA and H_2_O_2_ determination, gene expression analysis and enzyme activity analysis.

For gene expression analysis of different organs, well-uniformed rice seedlings were transplanted into ceramic pots (size: 60 cm in height and 30 cm in diameter) and grew under natural conditions. Different organs were sampled at the rapid grain-filing stage (about 10 days after flowering) and stored at −80°C pending assay.

### Gene Expression Analysis

Total RNA (from dry or imbibed seeds at indicated times or different plant organs) was extracted by using the RNeasy plant mini kit (Qiagen). DNase I-treated total RNA (2 mg) was denatured and subjected to reverse transcription using RevertAid™ first strand cDNA synthesis kit (100 units per reaction; Thermo Scientific). Transcript levels of each gene were measured by qRT-PCR using 7500 Real-Time PCR Systems (ABI) with iTaq™ Universal SYBR® Green Supermix (Bio-Rad). Gene expression was quantified at the logarithmic phase using the expression of the housekeeping *OsActin7* (LOC_Os11g06390) as an internal control. Three biological replicates were performed for each experiment. Primer sequences for qRT-PCR are shown in Table S1.

### Protein Blot Analysis

Rice seeds or leaves were homogenized in TEDM buffer (20 mM Tris/HCl, pH 7.5, 1 mM DTT, 5 mM EDTA, and 10 mM MgCl2) containing complete protease inhibitor cocktail (Roche). The homogenate was centrifuged at 6,000 g for 30 min at 4°C to remove cellular debris, and the supernatant was clarified by centrifugation at 5,000 g for 90 min at 4°C. The soluble proteins were separated by SDS–PAGE on a 10% gel and blotted onto Hybond-P membrane (Amersham Pharmacia Biotech). HSP82, OsPIP and eEF1α were used as loading control. The antibodies used in this experiment were bought from Beijing Protein Innovation (BPI).

### Fluorescent Localization of OsRACK1A

To determine the intracellular localization of OsRACK1A, *OsRACK1A* was cloned into pXZP008 vector to produce an *OsRACK1A-GFP* fusion construct driven by the maize ubiquitin promoter (*Ubi::OsRACK1A:GFP*). Rice protoplasts prepared from shoots were transformed with *Ubi::OsRACK1A:GFP* by polyethylene glycol treatment. The florescence signal was observed with an Inverted Microscope (Axio Observer A1, Zeiss) at 16 h after transformation.

### Quantitative Analyses of the Endogenous Levels of ABA and H_2_O_2_

Measurement of endogenous ABA levels of imbibed seeds was carried out based on the procedures described by Chen et al. (2006). Briefly, 30 grains of imbibed rice seeds were homogenized in 1 ml of distilled water and then shaken at 4°C overnight. The homogenates were centrifuged at 12,000 g for 10 min at 4°C and the supernatant were used directly for ABA assay. ABA analysis was carried out using the radioimmumoassay (RIA) method as described by Quarrie et al. [39]. The 450 µl reaction mixture contained 200 µl of phosphate buffer (pH 6.0), 100 µl of diluted antibody (Mac 252, Abcam) solution, 100 µl of [^3^H]ABA (about 8,000 c.p.m.) (Sigma) solution and 50 µl of crude extract. The mixture was then incubated at 4°C for 90 min and the bound radioactivity was measured in 50% saturated (NH_4_)_2_SO_4_-precipitated pellets with a liquid scintillation counter (LS6500, Beckman)

For measurement of endogenous H_2_O_2_ levels of imbibed seeds, 20 grains of seeds were homogenized in 500 µl phosphate buffer (20 mM K_2_HPO_4_, pH 6.5). After centrifugation, 50 µl of the supernatant was incubated with 0.2 U ml^−1^ horseradish peroxidase and 100 µM Amplex Red reagent (10-acetyl-3,7-dihydrophenoxazine) at room temperature for 30 min in darkness. The fluorescence was quantified using Epoch™ Microplate Reader (BioTek) (excitation at 560 nm and emission at 590 nm).

### Enzyme Activity Assay

Frozen seeds (20 grains) were homogenized on ice with 1.5 ml of 100 mM potassium phosphate buffer with protease inhibitor [1 mM EDTA, 10% (v/v) glycerol, 1% (v/v) Triton X 100, 7 mM β-mercaptoethanol, 100 mM NaF, 1 mM NaVO_3_, 1 mM Na_3_VO_4_, 10 mM Na_4_P_2_O_7_, 10 mM N-ethylmaleimide]. The homogenate was then centrifuged at 12,000 g for 10 min at 4 °C. A 100 µl aliquot of supernatant was taken into the assay buffer containing 50 mM Na-acetate, pH 5.2, 10 mM CaCl_2_, 2% boiled soluble potato starch, and incubated at 37 °C for 1 h. Then 100 µl of assay mixture was taken and mixed with 100 µl of reaction termination buffer (0.1 N NaOH) and 100 µl of 3,5-dinitrosalicylic acid (DNS) solution (40 mM DNS, 400 mM NaOH, 1 M K-Na tartrate) for 5 min at 100°C. After dilution with 700 µl of distilled water, the absorbance at 540 nm (A_540_) was measured, and the reducing power was evaluated with a standard curve obtained with glucose (0–20 µmol). One enzymatic unit is defined as the amount of enzyme releasing 1 mmol of glucose in 1 min.

### Statistical Analyses

Data were subjected to analysis of variance and psot-hoc comparisons (Duncan's multiple range test; *P*<0.05 significance level). We used SPPSS versions 13.0 software. Values presented are means ± SD of three replicates and significant difference is labeled as “*” in Figures.

## Results

### *OsRACK1A* Expression and Intracellular Localization

**Figure 1:**
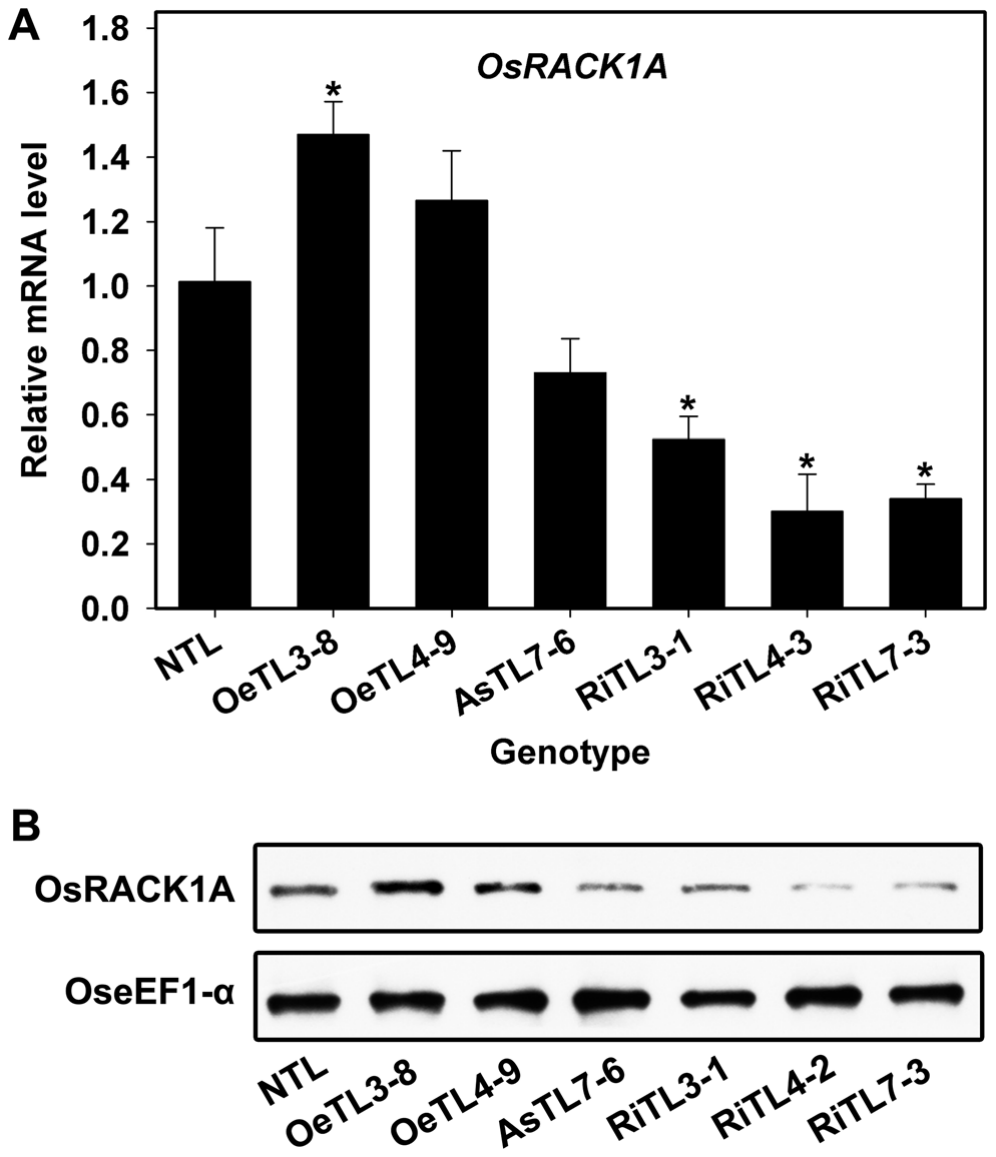
Expressional profile of OsRACK1A genes (A) and proteins (B) in selected transgenic rice lines. A, *OsRACK1A* expression was monitored in seedlings of wildtype (non-transgenic lines, NTL), *OsRACK1A* over-expressed transgenic lines (OeTL), anti-sense transgenic lines (AsTL) and RNA-interfered transgenic lines (RiTLs), respectively. Relative expression levels were calculated and normalized with respect to *OsActin7* (LOC_Os11g06390). Results shown are means of three biological replications±SE. Asterisks (*) indicate significant difference (*P*<0.05) between the levels of expression of transgenic lines compared with the wildtype (NTL). B, *OsRACK1A* expression was analyzed by incubating isolated proteins with polyclonal antibodies against OsRACK1A or OseEF1-α (as reference).

In rice, there are two RACK1 homologs, designated as OsRACK1A and OsRACK1B, which are approximately 80% similar to *Arabidopsis* RACK1 proteins at the amino acid level [26]. OsRACK1A and OsRACK1B share over 82.8% identity and 89.3% similarity at the amino acid level when aligned with Blast 2 Sequences [40]. Gene expression profile analysis indicated that *OsRACK1A* gene was highly expressed whereas *OsRACK1B* was lowly expressed and hardly detected in rice seeds during germination (Figure S1). Therefore, to explore the functions of *OsRACK1* in seed germination and seedling growth, we firstly generated a series of transgenic rice lines, of which the expression of *OsRACK1A* gene was either down-regulated using the RNA-interference or anti-sense techniques, or up-regulated using the Ubi-promoter [38]. The homozygous transgenic lines of the 6^th^ generation were used in this experiment. Figure 1 showed the *OsRACK1A* expression in imbibed seeds of the selected transgenic lines. As compared with that of wildtype (non-transgenic line, NTL), the transcript levels of *OsRACK1A* were 30∼50% higher in the over-expressed transgenic lines (OeTLs) and 50∼70% lower in RNA-interfered transgenic lines (RiTLs) or anti-sense transgenic lines (AsTLs) measured by quantitative RT-PCR (Figure 1A). Consistent with these results, western blot analysis using the OsRACK1A-specific antibody indicated that RACK1A protein was up-regulated in OeTLs and down-regulated in RiTLs (Figure 1B). Meanwhile, the transcript levels of *OsRACK1B* in RiTLs or AsTLs were also obviously lower than those in NTL or OeTLs (Figure S2).

**Figure 2:**
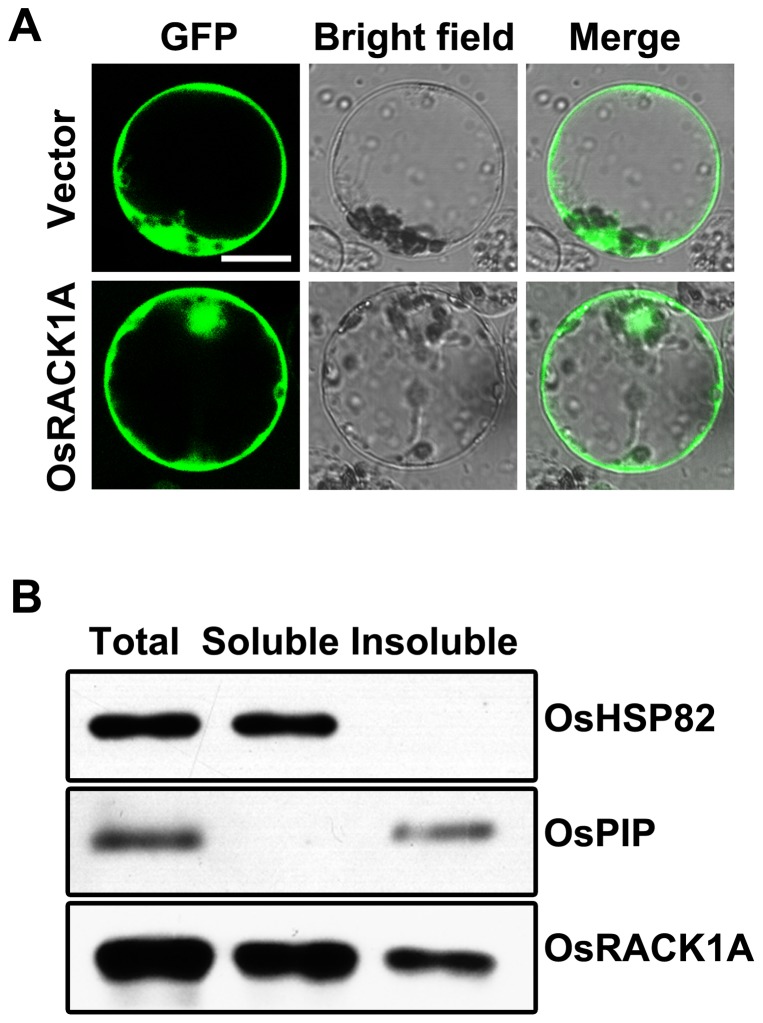
Intracellular localization of OsRACK1A. The intracellular localization of OsRACK1A was monitored with the fluorescence microscopy (A) and using immunoblotting analysis (B). Rice protoplasts were transformed with a vector containing *Ubi::OsRACK1A–GFP* or *Ubi::GFP* by PEG method and the florescence signal was observed with an Inverted Microscope (Axio Observer A1, Zeiss) at 16 h after transformation. For immunoblotting assay, the soluble and insoluble fractions of the cells were isolated by differential centrifugation and incubated with polyclonal antibodies against OsRACK1A or OsHSP82 (as reference) or OsPIP (as reference of insoluble protein).

The intracellular localization of OsRACK1A was also investigated using immunoblotting analysis and fluorescence microscopy. Rice protoplasts were transfected with a vector containing *Ubi*::*OsRACK1A–GFP or Ubi*::*GFP*. Fluorescence microscopic examination revealed that the GFP fluorescence was detected in the cytosol fraction as well as in the plasma membrane and in the nuclei (Figure 2A). To confirm the intracellular localization of OsRACK1A, we further prepared the soluble and insoluble fractions of the cells by differential centrifugation and incubated them with polyclonal antibodies against OsRACK1A. As shown in Figure 2B, heavy protein bands were detected in all fractions by western blot analysis. These results suggested that OsRACK1A was a soluble protein and may anchor in the plasma membrane by means of interacting with other molecules.

### Seed Germination and Its Responses to ABA and H_2_O_2_ in Different Transgenic Rice Lines

**Figure 3:**
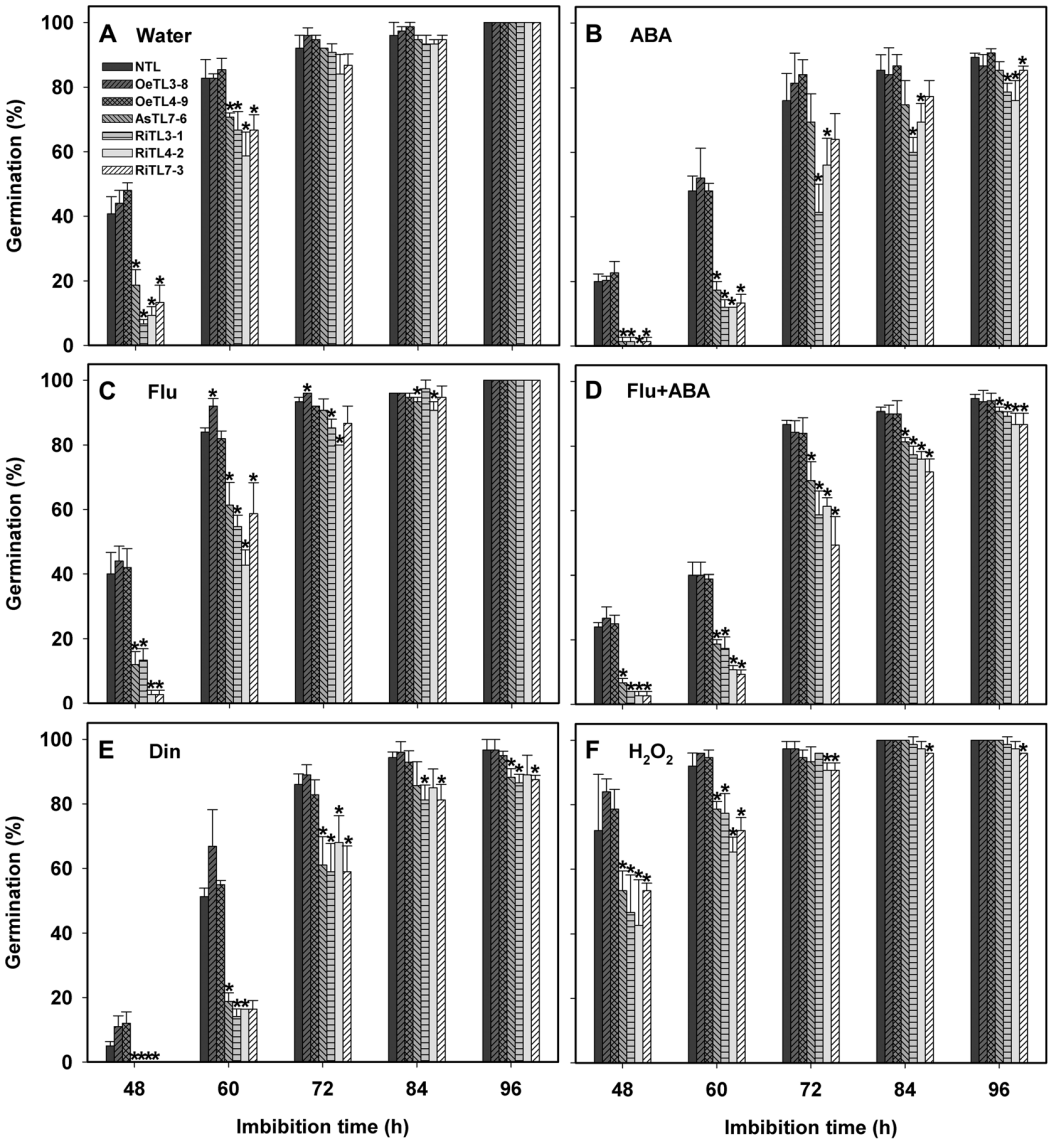
Effects of different treatments on seed germination of different transgenic lines. Sterilized seeds were germinated at 28°C on sterile filter papers in the petri dishes containing different concentrations of ABA (20 µM), fluridone (20 µM), diniconazole (100 µM) or H_2_O_2_ (20 mM). Germination (based on radicals >2 mm) was recorded at the indicated time points. Fifty seeds per genotype were used. Data shown are means of three biological replications±SE. Asterisks (*) indicate significant difference (*P*<0.05) between the seed germination of transgenic lines compared with the wildtype.

Quantitative RT-PCR (qRT-PCR) analysis of *OsRACK1A* expression patterns across various tissues and organs indicated that the highest level of *OsRACK1A* transcript was in seeds (Figure S1). Therefore, we wanted to examine whether *OsRACK1A* regulates seed germination. It is well known that seed germination is a multi-stage process and involves in complicated regulatory networks. To assess the roles of *OsRACK1* in controlling germination of rice, seeds of different transgenic rice lines were imbibed under standard germination conditions and germination kinetics was measured. Seed germination started after 36 h imbibition and then the germination rate was increased rapidly for NTLs. A significant delay in germination and a slowly increase in germination rate were observed for seeds of RiTLs (Figure 3A). ABA treatment delayed seed germination of all rice genotypes tested. However, germination of RiTLs or AsTLs was more sensitive to exogenous ABA treatment than those of NTLs and OeTLs (Figure 3B). These results suggested that *OsRACK1A* positively regulates seed germination in rice, as is the case in *Arabidopsis* reported earlier [28], and *OsRACK1A* may involve in ABA-mediated signaling during seed germination. In order to further explore the roles of endogenous ABA in controlling seed germination, fluridone, an inhibitor of ABA biosynthesis, and diniconazole, a potent competitive inhibitor of ABA catabolism, were applied and seed germination kinetics were measured. As shown in Figures 3C-E, fluridone treatment had no obvious effects on germination of all genotypes, as compared with the control treatment (Figure 3A). A similar result was also observed when comparison of germination was made between the combined treatment of fluridone with exogenous ABA and ABA treatment alone. However, diniconazole treatment significantly delayed seed germination of all genotypes, especially at the earlier stage of seed germination, i.e. within 48 h of imbibition. More severe delay of germination was observed in RiTLs and AsTLs lines (Figure 3E). We also observed that hypersensitivity of AsTLs and RiTLs to ABA inhibition of germination was ABA dosage-dependent (Figure S3). Taken together, these results indicated that the suppression of ABA catabolism rather than an enhancement of ABA biosynthesis may be the major factor for the delay of germination and *OsRACK1A* may exert its effect by controlling ABA metabolism.

It is well known that ABA treatment as well as adverse environmental factors induces production of H_2_O_2_ that may also involve in the regulation of seed germination [23]. However, it is largely unclear about the molecular mechanisms of H_2_O_2_ regulation on seed germination. In the present study, we assessed the effects of exogenous H_2_O_2_ treatment on seed germination of different transgenic rice lines. The result showed that exogenous H_2_O_2_ treatment significantly stimulated seed germination of all genotypes and after 48 h of imbibition, the germination rate reached approximately 80% for NTL and OeTLs and 45% for RiTLs and AsTLs (Figure 3F), as compared to about 40% of germination for NTL and OeTLs and 15% for RiTLs and AsTLs, and RiTLs and AsTLs were more sensitive to H_2_O_2_ promotion of seed germination. These results implied that H_2_O_2_ may also involve in the regulation of *OsRACK1A* on seed germination.

### ABA and H_2_O_2_ Interaction on Seed Germination of Different Transgenic Rice Lines

**Figure 4:**
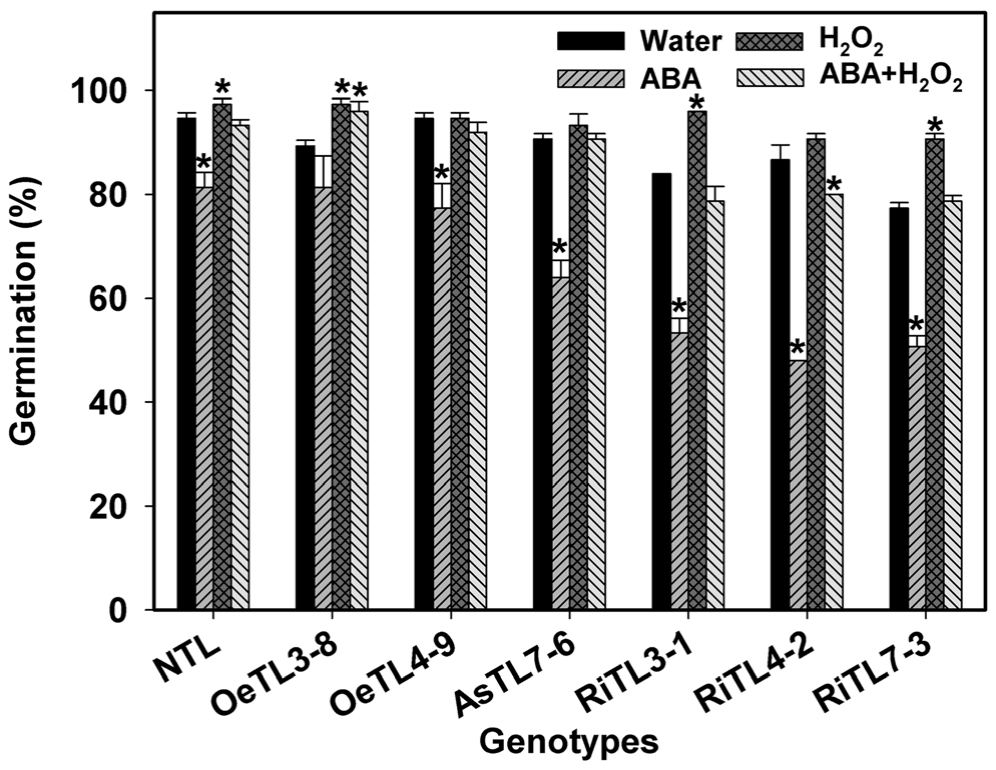
Effects of exogenous ABA and H_2_O_2_ on seed germination of different transgenic lines. Sterilized seeds were germinated at 28°C on sterile filter papers in the petri dishes containing different concentrations of ABA (20 µM) or H_2_O_2_ (20 mM) or both. Germination (based on radicals >2 mm) was recorded at 72 h after imbibition. Fifty seeds per genotype were used. Data shown are means of three biological replications±SE. Asterisks (*) indicate significant difference (*P*<0.05) between the seed germination of transgenic lines compared with the wildtype.

As described above, ABA had an inhibitory whereas H_2_O_2_ had a stimulated effect on seed germination. A lot of results have been shown that that ABA interacts with H_2_O_2_ in many processes, such as stomotal movement [41]. However, it is it is unclear how ABA interacts with H_2_O_2_ to regulate seed germination and what are their roles in *OsRACK1A*-mediating seed germination. Here we compared germination of different *OsRACK1A* transgenic rice lines when seeds were treated with ABA or H_2_O_2_ alone or both. As shown in Figure 4, ABA significantly suppressed and H_2_O_2_ stimulated seed germination. However, ABA inhibition on seed germination was either almost fully removed for NTL and OeTLs or significantly alleviated for RiTLs and AsTLs by H_2_O_2_ (Figure 4).

**Figure 5:**
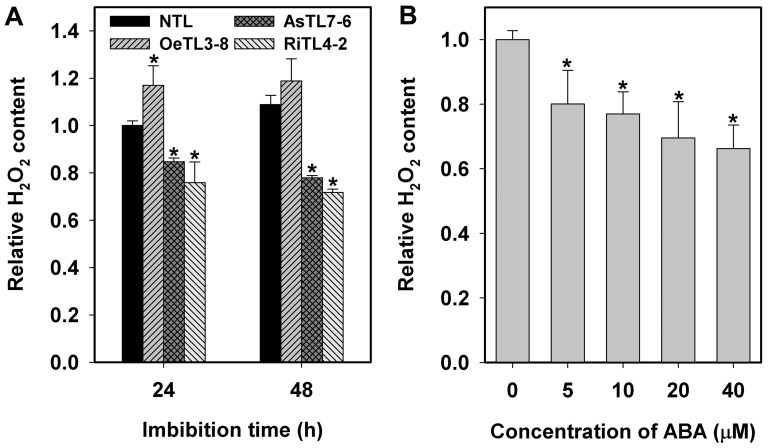
Comparison of relative H_2_O_2_ contents among different transgenic rice lines and the effect of ABA treatment on it in imbibed seeds. For measurement of endogenous H_2_O_2_ levels of imbibed seeds, twenty seeds were homogenized in 500 µl phosphate buffer (20 mM K_2_HPO_4_, pH 6.5). After centrifugation, 50 µl of the supernatant was incubated with 0.2 U ml^−1^ horseradish peroxidase and 100 µM Amplex Red reagent (10-acetyl-3,7-dihydrophenoxazine) at room temperature for 30 min in darkness. The fluorescence was quantified using EpochTM Microplate Reader (BioTek) (excitation at 560 nm and emission at 590 nm). Data shown are means of three biological replications±SE. Asterisks (*) indicate significant difference (*P*<0.05) between the relative levels of transgenic lines compared with the wildtype (A) or 0 µM ABA (B).

We further analyzed the H_2_O_2_ levels in different transgenic rice lines and the effects of ABA on H_2_O_2_ production in imbibed seeds. Great differences in H_2_O_2_ levels were detected in imbibed seeds of various transgenic rice lines (Figure 5A). As compared to NTL, the H_2_O_2_ level of OeTL was significantly higher and those of AsTL and RiTL were significantly lower, which may partially explain the difference in germination patterns among different transgenic rice lines. ABA treatment significantly inhibited the H_2_O_2_ production in germinating seeds, and this inhibition was ABA dosage-dependent (Figure 5B).

**Figure 6:**
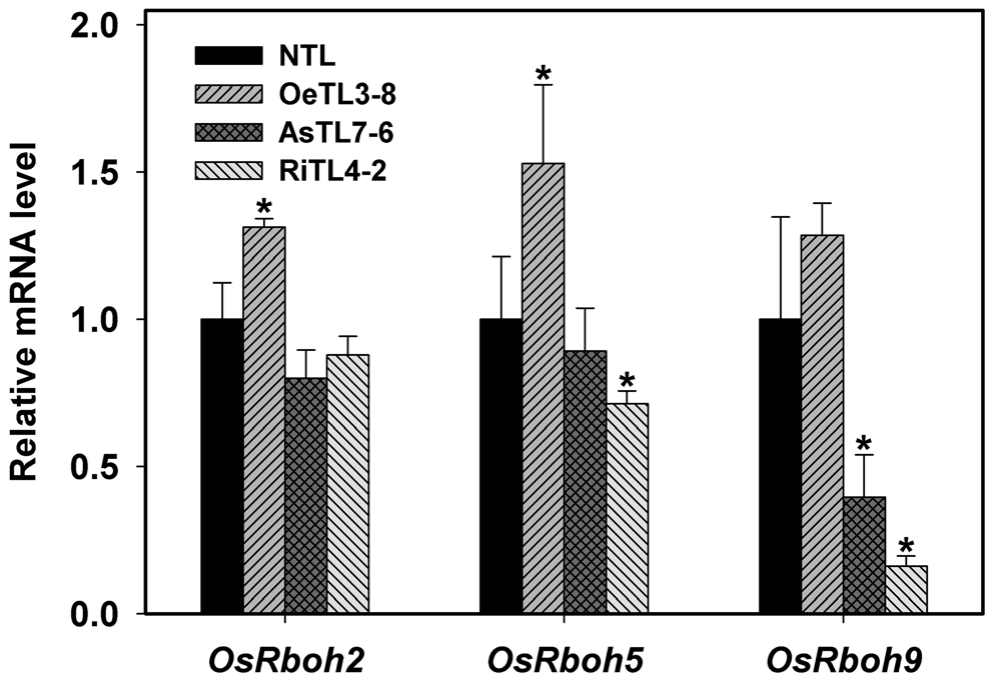
Expressional of *OsRbohs* in imbibed seeds of the selected transgenic rice lines. Expressions of *OsRbohs* were monitored in seeds of NTL, OeTL, AsTL and RiTL, respectively after 48 h of imbibition. Relative expression levels were measured by qRT-PCR and normalized with respect to *OsActin7* (LOC_Os11g06390). Results shown are means of three biological replications±SE. Asterisks (*) indicate significant difference (*P*<0.05) between the levels of expression of transgenic lines compared with the NTL.

NADPH oxidases have been proposed to be involved in ROS production during seed germination [42]. In rice genome, nine NADPH oxidases genes have been reported [43]. Our preliminary analysis has shown that only three genes, *OsRboh2*, *OsRboh5* and *OsRobh9*, expressed in imbibed seeds of rice. In order to clarify whether the expression of these genes is responsible for ROS (mainly H_2_O_2_) production and to explain the differences among various genotypes used in this experiment, the transcript levels of these three genes were determined in imbibed rice seeds using qRT-PCR. As shown in Figure 6, the transcripts of all these three genes could be detected in all genotypes examined. However, the transcript levels of all three genes were substantially higher in the imbibed seeds of OeTLs and significantly lower in those of AsTLs and RiTLs, as compared with those of NTL (Figure 6), which was consistent with the trends of endogenous H_2_O_2_ levels in these transgenic lines (Figure 5A). These results indicated that *OsRbohs* are responsible for the production of H_2_O_2_ in imbibed seeds and thus the seed germination.

### Changes in Endogenous ABA Levels during Germinating Process

**Figure 7:**
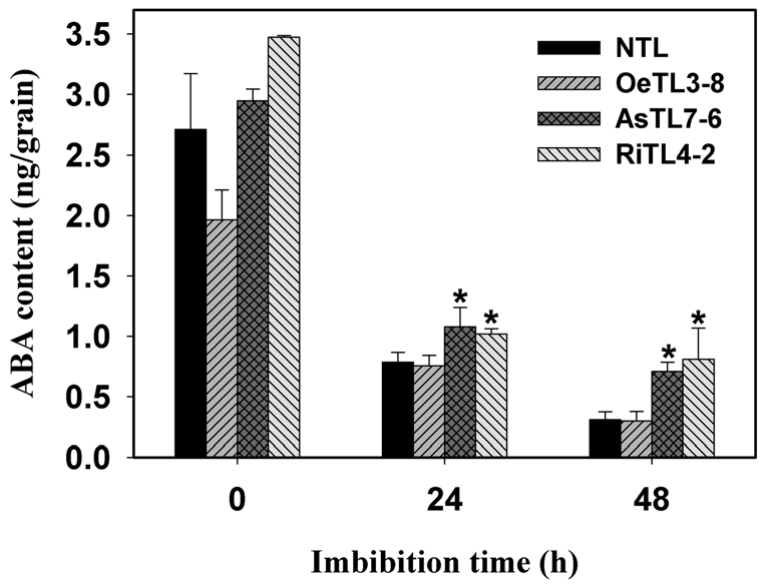
Comparison of endogenous ABA contents in imbibed seeds of different transgenic lines. ABA analysis was carried out using the radioimmumoassay (RIA) method. Thirty imbibed rice seeds were homogenized in 1 ml of distilled water and then shaken at 4°C overnight. The homogenates were centrifuged at 12,000 g for 10 min at 4°C and the supernatant were used directly for ABA assay. Values shown are means of three biological replications±SE. Asterisks (*) indicate significant difference (*P*<0.05) between the endogenous ABA contents of transgenic lines compared with the wildtype.

ABA is one of key hormones regulating seed germination. Our results had shown that *OsRACK1A* positively regulate seed germination. In order to investigate whether *OsRACK1* affects endogenous ABA levels, we analyzed the endogenous ABA levels in germinating seeds of different *OsRACK1A* transgenic lines. Seeds of different transgenic rice lines were imbibed for different times and ABA levels were detected by radioimmumoassay (RIA) method as described by Quarrie et al. [39]. As shown in Figure 7, significant decreases in endogenous ABA levels were observed with imbibition time proceeded and there were significant differences in ABA levels among different transgenic rice lines. ABA levels in imbibed seeds of AsTLs and RiTLs were significantly higher than those of NTL and OeTLs, which may, at least in part, explain the slow initiation and delay of seed germination of AsTLs and RiTLs.

### Changes of Amylase Activity and Amylase Gene Expression in Imbibed Seeds of Different Transgenic Rice Lines

**Figure 8:**
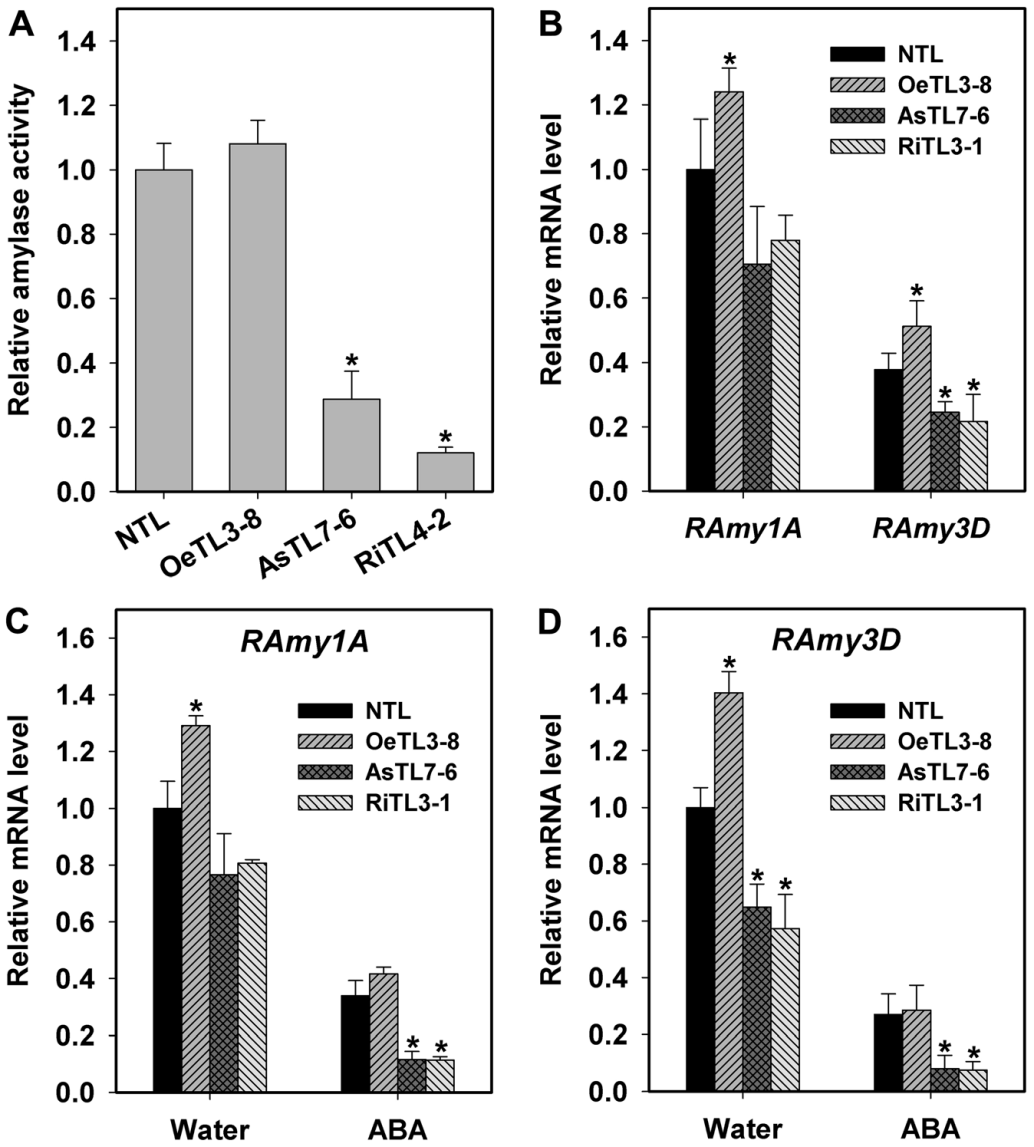
Comparisons of total amylase activity and of expression of two key amylase genes, *RAmy1A* and *RAmy3D*, in imbibed seeds of different transgenic lines and the effects of ABA. A, For amylase activity assay, frozen seeds (20 grains) were homogenized on ice and 100 µl aliquot of supernatant was taken into the assay after centrifugation. The reaction product was monitored using 3,5-dinitrosalicylic acid (DNS) method. B, C and D, Expressions of *RAmy1A* and *RAmy3* were monitored in seeds of NTL, OeTL, AsTL and RiTL, respectively after 48h of imbibition in water or in ABA (20 µM). Relative expression levels were measured by qRT-PCR and normalized with respect to *OsActin7* (LOC_Os11g06390). Results shown are means of three biological replications±SE. Asterisks (*) indicate significant difference (*P*<0.05) between the levels of expression of transgenic lines compared with the NTL.

Seed germination is a complex physiological and biochemical process and involves the degradation of stored compounds and re-synthesis of new compounds necessary for cell growth and development. Because in mature rice seeds, about 80% of stored carbohydrate is starch, starch degradation is necessary for germination and early growth and development of seedling by providing both energy and carbon cytoskeleton. Amylase is a key enzyme catalyzing starch degradation of germinating seeds. Therefore, understanding the molecular mechanism of amylase in controlling seed germination is of predominant importance in agronomic practice. In rice genome, there are 12 genes putatively encoding alpha–amylases, but only two of them have been identified in rice seeds, *RAmy1A* and *RAmy3D* (unpublished). In the present study, seeds of different *OsRACK1A* transgenic lines imbibed for 24 hours were sampled to analyze the amylase activity and the expression of its encoding genes. A great differences were detected in amylase activity among different transgenic rice lines and the amylase activity of AsTLs and RiTLs was only 15∼30% of that NTL and OeTLs (Figure 8A). Among two amylase genes expressed in seeds, the transcript level of *RAmy1A* was about two times higher than that of *RAmy3D*. Furthermore, compared to those in the NTL, the expression of both *RAmy1A* and *RAmy3D* was much lower in AsTLs and RiTLs and much higher in OeTLs (Figure 8B). ABA had significant inhibitory effects on the expression of both *RAmy1A* and *RAmy3D* (Figure 8C and D). Taken together, these results suggested that *OsRACK1A* positively regulates germination through up-regulating amylase activity and the expression of *RAmy1A* and *RAmy3D* in rice seeds.

## Discussion

RACK1 is a strongly conserved and scaffolding protein and expresses ubiquitously. It is well recognized that RACK1 is involved in multiple signaling pathways, including growth and development and responses to external environmental cues [28],[34]. However, as compared with the progresses in the field of mammalian RACK1, our understanding of the functions and molecular mechanisms of plant RACK1 is still in its infancy. We and several other research teams have studied the roles of RACK1 in plant growth and development and in the responses to environmental cues and found that RACK1 is involved in regulating cell proliferation and elongation, inoculation and the responses to plant hormones and environmental factors [28],[35], [37], [44], [45]. In rice genome, there are two RACK1 homologous genes, *OsRACK1A* and *OsRACK1B*. Nakashima et al. [34] reported that RACK1A functions in rice innate immunity by interacting with multiple proteins in the Rac1 immune complex. Our study has shown that, although OsRACK1A and OsRACK1B are high similarity each other, the expression patterns are obviously different and *OsRACK1B* transcript levels are always significantly lower than those of *OsRACK1A*, especially in roots and mature seeds (Figure S1). The highest *OsRACK1A* transcript level and the lowest *OsRACK1B* transcript level are founded in mature seeds. These results imply that *OsRACK1A* is a major gene which may play an important role in controlling seed development and germination. In present study, by generating several transgenic rice lines, of which *OsRACK1A* was either down-regulated or up-regulated, and analyzing the roles of *OsRACK1A* in seed germination and its possible mechanisms, we show that *OsRACK1A* positively regulates seed germination through its enhancement of ABA catabolism and stimulation of H_2_O_2_ production, and that ABA interacts with H_2_O_2_ on the regulation of seed germination. As compared with that of wildtype rice (NTL) seeds, when *OsRACK1A* is down-regulated, seed germination is significantly delayed, and over-expression of *OsRACK1A* has, although not obvious, a stimulating effect on germination. This case is very similar to that in *Arabidopsis* seed germination [28]. However, we currently know little about how RACK1 regulates the seed germination. In other words, what are the component(s) in RACK1-mediated signaling during germination? Because ABA is well-known to involve in maintenance of seed dormancy and inhibition of seed germination, it is reasonable to assume that the inhibition of germination as a result of *OsRACK1A* down-regulation may be related to ABA. This assumption is verified in this study. We show that, although exogenous ABA treatment inhibits germination of all genotypes used, this inhibition of germination of *OsRACK1A* down-regulated transgenic rice seeds is much more sensitive to ABA than those of non-transgenic line and of *OsRACK1A* over-expressed transgenic lines (Figures 3, S3). This phenomenon suggests that *OsRACK1A* is a negative regulator of ABA response in seed germination of rice, which is very similar to our previous results that RACK1 negatively regulates the responses of seed germination, cotyledon greening and root growth to ABA treatment in *Arabidopsis* [35]. Furthermore, analysis on the endogenous ABA levels in dry and imbibed seeds shows that the endogenous ABA levels of both dry and imbibed seeds of *OsRACK1A* down-regulated transgenic rice lines are significantly higher than those in seeds of wildtype and *OsRACK1A* over-expressed transgenic lines (Figure 7). These results may explain well why the seed germination of *OsRACK1A* down-regulated transgenic rice lines is delayed under the same conditions.

In *Arabidopsis thaliana*, up-regulation of ABA biosynthesis is suggested as one of the possible mechanisms mediating the glucose-induced delay in seed germination, because the expression of ABA biosynthesis-related genes, such as *ABA2* and *NCED3* increases in responding to glucose signaling [46],[47]. However, our results show that higher endogenous ABA levels and the delay of seed germination of *OsRACK1A* down-regulated transgenic rice lines are not due to an enhancement of ABA biosynthesis because treatment with fluridone, a potent inhibitor of ABA biosynthesis, produces no obvious signs of enhancement of the germination rate. However, treatment with ABA catabolic inhibitor, diniconazole, dramatically decreased the seed germination rate, as compared with those of control (water) treatment (Figure 3). These results suggest that higher endogenous ABA levels of seeds of *OsRACK1A* down-regulated transgenic rice lines are mainly due to the decrease of ABA catabolism, and thus result in a delay of seed germination. A similar situation was also observed in rice using another experimental system, where, Zhu et al. [2] found that glucose-induced delay of seed germination is a result of the suppression of ABA catabolism rather than any enhancement of ABA biosynthesis during rice seed germination. However, at present, we still know little about the molecular mechanisms of *OsRACK1A* regulation on ABA metabolism during seed germination and it deserves to study further.

ROS change dramatically during seed development and germination, implying that ROS play roles in seed dormancy alleviation, after-ripening, and germination [22],[48]. Although ROS are long considered as hazardous molecules, many recent studies have provided physiological and genetic evidence that ROS may also function as messengers or transmitters of environmental cues during seed germination. There are many reports indicated that seed germination and ROS accumulation appear to be linked, and that seed germination success may be closely associated with internal ROS contents and the activities of ROS-scavenging systems in species such as *Arabidopsis thaliana* [21],[49], sunflower [22], wheat [50], cress [51] and barley [12], soybean [20], rice [24]. Our results present here suggest that *OsRACK1A* may also regulate seed germination by mediating ROS production in imbibed seeds. This finding is supported by several lines of evidence. Externally supplied ROS in the form of H_2_O_2_ stimulates seed germination of all genotypes used and again the germination of *OsRACK1A* down-regulated transgenic rice lines is more sensitive to H_2_O_2_ (Figure 3), which is consistent with the cases in several other plant species [20],[21],[52]. Furthermore, comparison of the seed germination rate and the endogenous H_2_O_2_ levels in imbibed seeds among different rice genotypes, a lower germination rate is paralleled by a lower endogenous level of H_2_O_2_. For example, after imbibition for 48 h, the germination rate is 40% and around 12% for wildtype and *OsRACK1A* down-regulated transgenic rice lines, respectively, whereas the relative endogenous level of H_2_O_2_ is 1.14 and 0.78 for wildtype and *OsRACK1A* down-regulated transgenic rice lines, respectively (Figures 3, 5A). These results imply that *OsRACK1A* may also control H_2_O_2_ production of imbibed seeds, in spite that its molecular mechanism is still unknown. Pharmacological and molecular studies have shown that *Rbohs* genes and NADPH oxidase activity are potentially important to the germination process [21]. Treatment with diphenyleneiodonium (DPI), an inhibitor of *Rbohs*, blocked seed germination, suggesting that internal generation of ROS by *Rbohs* was needed for seed germination [53]. Seeds of the *atrbohB* mutant fail to after-ripen due to its blocked ROS production [53]. Our preliminary studies indicated that only three of nine *Rbohs* genes, i.e. *Rboh2*, *Rboh5* and *Rboh9*, were expressed in imbibed rice seeds. Our results present here show that the transcript levels of these three *Rbohs* genes in imbibied seeds of *OsRACK1A* down-regulated transgenic rice lines are significantly lower than those of wildtype, whereas the highest transcript levels are detected in *OsRACK1A* up-regulated transgenic rice lines. These results are consistent with the results of internal H_2_O_2_ levels (Figure 5). Taken together, we suggest that the positive control of *OsRACK1A* on seed germination is, at least in part, through up-regulating *Robhs* gene expression, as the results, stimulating internal H_2_O_2_ production.

There are many reports on the interaction of ROS with plant hormones in several physiological and molecular processes in plants. Involvement of ROS as second messengers in ABA-mediated stomatal movement has been well documented [41]. Generally, ABA induces ROS production in the guard cells as well as in non-seed tissues under adverse conditions. However, in seed physiology, ABA reduces ROS production in germinating seeds (Figures 5B),[24]. On the other hand, exogenous H_2_O_2_ treatment can mostly recover seed germination inhibited by ABA (Figure 4). Taking it into consideration that a rapid decrease in endogenous ABA level and gradual increase in endogenous H_2_O_2_ level occur during seed germination, it is understandable that there must be a fine mechanism maintaining a fine balance between ABA and H_2_O_2_ levels for germination success. This topic is deserved to further investigation.

**Figure 9:**
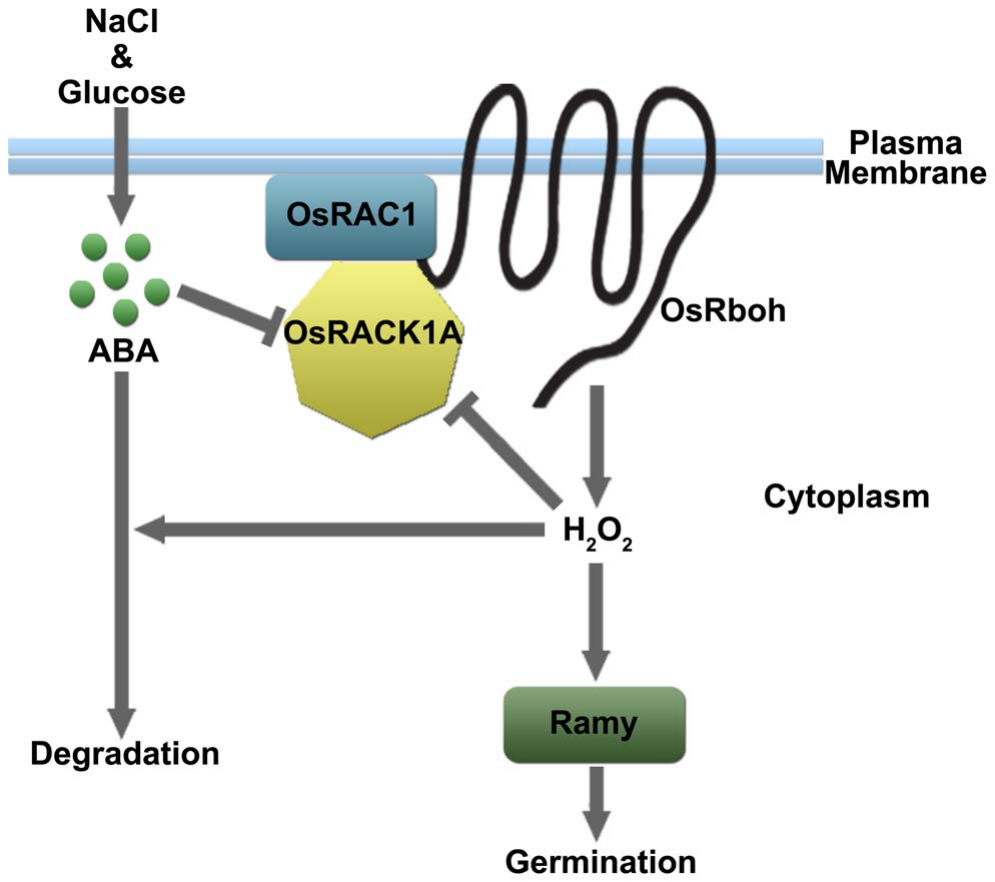
A working model showing proposed molecular steps of OsRACK1A-mediated signal transduction pathway. This model presents a schematic of OsRACK1A and other signal molecules involved in the proposed signal transduction pathway during seed germination. The results presented in this work combined with the results reported by Nakashima et al. (2008) to support this signal transduction. Under normal germination conditions, cytosol OsRACK1A moved towards plasma membrane and interacted with OsRAC1 to form a complex. The formed complex then activated downstream OsRboh, and as a result, stimulated H_2_O_2_ production and seed germination. When responding to external stimuli, which may induce ABA accumulation (Figure S4; Zhu et al, 2009), excess ABA inhibited *OsRACK1* expression and blocked the formation of OsRACK1A-OsRAC1 complex, and as a consequence, suppressed the expression of *OsRbohs* and the production of H_2_O_2_ (Figure 5). Excess H_2_O_2_ in return suppressed *OsRACK1* expression to avoid its hazardous effect (Figure S5). In addition, H_2_O_2_ may also stimulate ABA catabolism and alleviated the inhibitory effect on seed germination.

In summary, it is concluded that *OsRACK1A* positively regulates seed germination by controlling endogenous levels of ABA and ROS and their interaction. Under normal germination conditions, cytosol OsRACK1A moved towards plasma membrane and interacted with OsRAC1 to form a complex [34]. The formed complex then activated downstream OsRboh, and as a result, stimulated H_2_O_2_ production and seed germination. When responding to external stimuli, which may induce ABA accumulation (Figure S4),[2], excess ABA inhibited *OsRACK1* expression and blocked the formation of OsRACK1A-OsRAC1 complex, and as a consequence, suppressed the expression of *OsRbohs* and the production of H_2_O_2_ (Figure 5). Excess H_2_O_2_ in return suppressed *OsRACK1* expression to avoid its hazardous effect (Figure S5). In addition, H_2_O_2_ may also stimulate ABA catabolism and alleviated the inhibitory effect on seed germination (Figure 9).

## Supporting Information

Figure S1 **Quantitative RT-PCR analysis of** ***OsRACK1A*** **and** ***OsRACK1B*** **expression in rice tissues.** Total RNA isolated from root, stem, leaf, panicle, dry seed or seedling (2 weeks) was used as template for qRT-PCR. Relative expression levels were calculated and normalized with respect to OsActin7 (LOC_Os11g06390). Results shown are means of three biological replications±SE.
(TIF)

Figure S2 **Expressional profile of** ***OsRACK1B*** **in selected transgenic rice lines.** *OsRACK1B* expression was monitored in seedlings of wildtype (non-transgenic lines, NTL), *OsRACK1A* over-expressed transgenic lines (OeTL), anti-sense transgenic lines (AsTL) and RNA-interfered transgenic lines (RiTLs), respectively. Relative expression levels were calculated and normalized with respect to *OsActin7* (LOC_Os11g06390). Results shown are means of three biological replications±SE.
(TIF)

Figure S3 **Effects of different concentration of ABA treatments on seed germination of different transgenic lines.** A. Morphology of germinating seeds in the different concentration of ABA (0, 5, 10 and 20 µM) treatment for 24 hours. B. Seed germination rates under 0, 5, 10 and 3 µM ABA treatment for 24 hours. Each value is the mean ± standard error of at least 50 seeds. Asterisks (*) indicate significant difference (P<0.05) between the seed germination of transgenic lines compared with the wildtype.
(TIF)

Figure S4 **Effects of different concentrations of glucose, NaCl and H_2_O_2_ on seed germination of different transgenic lines.** Sterilized seeds were germinated at 28°C on sterile filter papers in the petri dishes containing different concentrations of glucose (0, 1, 3 and 6 mM), NaCl (0, 50, 100 and 200 mM) or H_2_O_2_ (0, 10, 20 and 40 mM). Germination (based on radicals >2 mm) was recorded at the indicated time points. Fifty seeds per genotype were used. Data shown are means of three biological replications±SE. Asterisks (*) indicate significant difference (P<0.05) between the seed germination of transgenic lines compared with the wildtype.
(TIF)

Figure S5 **Effects of ABA and H_2_O_2_ on expressional profile of OsRACK1A genes (A) and proteins (B) in selected transgenic rice lines.** Two-week-old seedlings were treatment with different concentration of ABA (5, 10, 20 and 40 µM) or H_2_O_2_ (10, 20 and 40 mM) for 24 h before RNA or protein extraction. A. Relative gene expression levels were calculated and normalized with respect to *OsActin7* (LOC_Os11g06390). B. OsRACK1A expression was analyzed by incubating isolated proteins with polyclonal antibodies against OsRACK1A or OseEF1-α (as reference).
(TIF)

Table S1 Gene specific primers used in quantitative real-time PCR (qRT-PCR).
(DOCX)

## References

[1] Finkelstein R, Reeves W, Ariizumi T, Steber C (2008) Molecular aspects of seed dormancy. Annu Rev Plant Biol 59: 387–415. 18257711 10.1146/annurev.arplant.59.032607.092740

[2] Zhu G, Ye N, Zhang J (2009) Glucose-induced delay of seed germination in rice is mediated by the suppression of ABA catabolism rather than an enhancement of ABA biosynthesis. Plant Cell Physiol 50(3): 644–651. 19208695 10.1093/pcp/pcp022

[3] He D, Han C, Yao J, Shen S, Yang P (2011) Constructing the metabolic and regulatory pathways in germinating rice seeds through proteomic approach. Proteomics 11: 2693–2713. 21630451 10.1002/pmic.201000598

[4] Diaz-Vivancos P, Barba-Espín G, Hernández JA (2013) Elucidating hormonal/ROS networks during seed germination: insights and perspectives. Plant Cell Rep. doi: 10.1007/s00299-013-1473-7. 10.1007/s00299-013-1473-7 23812175

[5] Nanogaki H, Basel GW, Bewley JD (2010) Germination-still a mystery. Plant Sci 179: 574–581.

[6] Bassel GW, Fung P, Chow TFF, Foong JA, Provart NJ, et al (2008) Elucidating the germination transcriptional program using small molecules. Plant Physiol 147: 143–155. 18359847 10.1104/pp.107.110841 PMC2330302

[7] Bassel GW, Lan H, Glaab E, Gibbs DJ, Gerjets T, et al (2011) Genome-wide network model capturing seed germination reveals coordinated regulation of plant cellular phase transitions. Proc Natl Acad Sci USA 108: 9709–9714. 21593420 10.1073/pnas.1100958108 PMC3111290

[8] Belmonte MF, Kirkbride RC, Stone SL, Pelletiera JM, Bui AQ, et al (2013) Comprehensive developmental profiles of gene activity in regions and subregions of the Arabidopsis seed. Proc Natl Acad Sci USA 14: E435–E444. 10.1073/pnas.1222061110 PMC3562769 23319655

[9] Gallardo K, Job C, Groot SP, Puype M, Demol H, et al (2001) Proteomic analysis of Arabidopsis seed germination and priming. Plant Physiol 126: 835–848. 11402211 10.1104/pp.126.2.835 PMC111173

[10] Howell KA, Narsai R, Carroll A, Ivanova A, Lohse M, et al (2009) Mapping metabolic and transcript temporal switches during germination in rice highlights specific transcription factors and the role of RNA instability in the germination process. Plant Physiol 149: 961–980. 19074628 10.1104/pp.108.129874 PMC2633829

[11] Yang P, Li X, Wang X, Chen H, Chen F, et al (2007) Proteomic analysis of rice (Oryza sativa) seeds during germination. Proteomics 7: 3358–3368. 17849412 10.1002/pmic.200700207

[12] Bahin E, Bailly C, Sotta B, Kranner I, Corbineau F, et al (2011) Crosstalk between reactive oxygen species and hormonal signalling pathway regulates grain dormancy in barley. Plant Cell Environ 34: 980–993. 21388415 10.1111/j.1365-3040.2011.02298.x

[13] Barba-Espín G, Diaz-Vivancos P, Clemente-Moreno MJ, Albacete A, Faize L, et al (2010) Interaction between hydrogen peroxide and plant hormones during germination and the early growth of pea seedlings. Plant Cell Environ 33: 981–994. 20102539 10.1111/j.1365-3040.2010.02120.x

[14] Kim ST, Kang SY, Wang Y, Kim SG, Hwang DH, et al (2008) Analysis of embryonic proteome modulation by GA and ABA from germinating rice seeds. Proteomics 8: 3577–3587. 18686304 10.1002/pmic.200800183

[15] Finkelstein R, Gampala SS, Rock CD (2002) Abscisic acid signaling in seeds and seedlings. Plant Cell 14 Suppl: S15–45. 12045268 10.1105/tpc.010441 PMC151246

[16] Holdsworth MJ, Bentsink L, Soppe WJ (2008) Molecular networks regulating Arabidopsis seed maturation, after-ripening, dormancy and germination. New Phytol 179: 33–54. 18422904 10.1111/j.1469-8137.2008.02437.x

[17] Okamoto M, Tatematsu K, Matsui A, Morosawa T, Ishida J, et al (2010) Genome-wide analysis of endogenous abscisic acid-mediated transcription in dry and imbibed seeds of Arabidopsis using tiling arrays. Plant J 62: 39–51. 20088898 10.1111/j.1365-313X.2010.04135.x

[18] Bailly C (2004) Active oxygen species and antioxidants in seed biology. Seed Sci Res 14: 93–107.

[19] Bailly C, El-Maarouf-Bouteau H, Corbineau F (2008) From intracellular signaling networks to cell death: the dual role of reactive oxygen species in seed physiology. CR Biol 331: 806–814. 10.1016/j.crvi.2008.07.022 18926495

[20] Ishibashi Y, Koda Y, Zheng S-H, Yuasa T, Iwaya-Inoue M (2013) Regulation of soybean seed germination through ethylene production in response to reactive oxygen species. Ann Bot 111: 95–102. 23131300 10.1093/aob/mcs240 PMC3523653

[21] Liu Y, Ye N, Liu R, Chen M, Zhang J (2010) H2O2 mediates the regulation of ABA catabolism and GA biosynthesis in Arabidopsis seed dormancy and germination. J Exp Bot 61: 2979–2990. 20460363 10.1093/jxb/erq125 PMC2892143

[22] Oracz K, El Maarouf-Bouteau H, Farrant JM, Cooper K, Belghazi M, et al (2007) ROS production and protein oxidation as a novel mechanism for seed dormancy alleviation. Plant J 50: 452–465. 17376157 10.1111/j.1365-313X.2007.03063.x

[23] Oracz K, El Maarouf-Bouteau H, Kranner I, Bogatek R, Corbineau F, et al (2009) The mechanisms involved in seed dormancy alleviation by hydrogen cyanide unravel the role of reactive oxygen species as key factors of cellular signalling during germination. Plant Physiol 150: 494–505. 19329562 10.1104/pp.109.138107 PMC2675718

[24] Ye N, Zhu G, Liu Y, Zhang A, Li Y, et al (2012) Ascorbic acid and reactive oxygen species are involved in the inhibition of seed germination by abscisic acid in rice seeds. J Exp Bot 63: 1809–1822. 22200664 10.1093/jxb/err336 PMC3295380

[25] Adams DR, Ron D, Kiely PA (2011) RACK1, A multifaceted scaffolding protein: Structure and function. Cell Comm Signal 9(22): 823–830. 10.1186/1478-811X-9-22 PMC3195729 21978545

[26] Guo J, Chen JG, Liang JS (2007) RACK1, a Versatile Scaffold Protein in Plant? Internl J Plant Dev Biol 1: 95–105.

[27] Smith TF, Gaitatzes C, Saxena K, Neer EJ (1999) The WD repeat: a common architecture for diverse functions. Trends Biochem Sci 24: 181–185. 10322433 10.1016/s0968-0004(99)01384-5

[28] Chen JG, Ullah H, Temple B, Liang J, Guo J, et al (2006) RACK1 mediates multiple hormone responsiveness and developmental processes in Arabidopsis. J Exp Bot 57: 2697–2708. 16829549 10.1093/jxb/erl035

[29] Chen S, Dell EJ, Lin F, Sai J, Hamm HE (2004) RACK1 regulates specific functions of Gbetagamma. J Biol Chem 279: 17861–17868. 14963031 10.1074/jbc.M313727200

[30] Guo J, Wang S, Valerius O, Hall H, Zeng Q, et al (2011) Involvement of Arabidopsis RACK1 in protein translation and its regulation by abscisic acid. Plant Physiol 155: 370–383. 21098678 10.1104/pp.110.160663 PMC3075769

[31] Ishida S, Takahashi Y, Nagata T (1993) Isolation of cDNA of an auxin-regulated gene encoding a G protein beta subunit-like protein from tobacco BY-2 cells. Proc Natl Acad Sci USA 90: 11152–11156. 8248221 10.1073/pnas.90.23.11152 PMC47940

[32] Ishida S, Takahashi Y, Nagata T (1996) The mode of expression and promoter analysis of the arcA gene, an auxin-regulated gene in tobacco BY-2 cells. Plant Cell Physiol 37: 439–448. 8759913 10.1093/oxfordjournals.pcp.a028965

[33] Iwasaki Y, Komano M, Ishikawa A, Sasaki T, Asahi T (1995) Molecular cloning and characterization of cDNA for a rice protein that contains seven repetitive segments of the Trp-Asp forty-amino-acid repeat (WD-40 repeat). Plant Cell Physiol 36: 505–510. 7757340 10.1093/oxfordjournals.pcp.a078786

[34] Nakashima A, Chen L, Thao NP, Fujiwara M, Wong HL, et al (2008) RACK1 functions in rice innate immunity by interacting with the Rac1 immune complex. Plant Cell 20: 2265–2279. 18723578 10.1105/tpc.107.054395 PMC2553611

[35] Guo J, Wang J, Xi L, Huang WD, Liang J, et al (2009) RACK1 is a negative regulator of ABA responses in Arabidopsis. J Exp Bot 60(13): 3819–3833. 19584117 10.1093/jxb/erp221 PMC2736894

[36] Islas-Flores T, Guillén G, Islas-Flores I, Román-Roque CS, Sánchez F, et al (2009) Germination behavior, biochemical features and sequence analysis of the RACK1/arcA homolog from Phaseolus vulgaris. Physiol Plant 137(3): 264–280. 19832940 10.1111/j.1399-3054.2009.01280.x PMC3376080

[37] Islas-Flores T, Guillén G, Sánchez F, Villanueva MA (2012) Changes in RACK1 expression induce defects in nodulation and development in Phaseolus vulgaris. Plant Sig Behav 7: 1–3. 10.4161/psb.7.1.18485 PMC3357353 22301979

[38] Li DH, Liu H, Yang YL, Zhen PP, Liang JS (2008) Down-Regulated Expression of RACK1 Gene by RNA Interference Enhances Drought Tolerance in Rice. Chin J Rice Sci 22: 447–453.

[39] Quarrie SA, Whitford PN, Appleford NEJ, Wang TL, Cook SK, et al (1988) A monoclonal antibody to (S)-abscisic acid: its characterization and use in a radioimmunoassay for measuring abscisic acid in crude extracts of cereal and lupin leaves. Planta 173: 330–339. 24226540 10.1007/BF00401020

[40] Li DH, Zhang DP, Cao DD, Liang JS (2011) Advances in Plant RACK1 studies. Chin Bull Bot 46 (2): 224–232.

[41] Wang P, Song CP (2008) Guard-cell signaling for hydrogen peroxide and abscisic acid. New Phytol 178: 703–718. 18373649 10.1111/j.1469-8137.2008.02431.x

[42] Sagi M, Fluhr R (2006) Production of reactive oxygen species by plant NADPH oxidases. Plant Physiol 141: 336–340. 16760484 10.1104/pp.106.078089 PMC1475462

[43] Liu J, Zhou J, Xing D (2012) Phosphatidylinositol 3-Kinase Plays a Vital Role in Regulation of Rice Seed Vigor via Altering NADPH Oxidase Activity. PLoS ONE 7(3): e33817 doi:10.1371/journal.pone.0033817 22448275 10.1371/journal.pone.0033817 PMC3309022

[44] Kundu N, Dozier U, Deslandes L, Somssich IE, Ullah H (2013) Arabidopsis scaffold protein RACK1A interacts with diverse environmental stress and photosynthesis related proteins. Plant Signal Behav 8: e24012. 23435172 10.4161/psb.24012 PMC3906143

[45] Wamaitha MJ, Yamamoto R, Wong HL, Kawasaki T, Kawano Y, et al (2012) OsRap2.6 transcription factor contributes to rice innate immunity through its interaction with Receptor for Activated Kinase-C 1 (RACK1). Rice 5: 35 doi:10.1186/1939-8433-5-35 24280008 10.1186/1939-8433-5-35 PMC4883712

[46] Cheng WH, Endo A, Zhou L, Penney J, Chen HC, et al (2002) A unique short-chain dehydrogenase/reductase in Arabidopsis glucose signaling and abscisic acid biosynthesis and functions. Plant Cell 14: 2723–2743. 12417697 10.1105/tpc.006494 PMC152723

[47] Chen Y, Ji F, Xie H, Liang J, Zhang J (2006) The regulator of G protein signaling proteins involved in sugar and abscisic acid signaling in Arabidopsis seed germination. Plant Physiol 140: 302–310. 16361523 10.1104/pp.105.069872 PMC1326052

[48] El-Maarouf-Bouteau H, Bailly C (2008) Oxidative signaling in seed germination and dormancy. Plant Signal Behav 3: 175–182. 19513212 10.4161/psb.3.3.5539 PMC2634111

[49] Leymarie J, Vitkauskaité G, Hoang HH, Gendreau E, Chazoule V, et al (2012) Role of reactive oxygen species in the regulation of Arabidopsis seed dormancy. Plant Cell Physiol 53(1): 96–106. 21937678 10.1093/pcp/pcr129

[50] Ishibashi Y, Yamamoto K, Tawaratsumida T, Yuasa T, Iwaya-Inoue M (2008) Hydrogen peroxide scavenging regulates germination ability during wheat (Triticum aestivum L.) seed maturation. Plant Signal Behav 3(3): 183–188. 19513213 10.4161/psb.3.3.5540 PMC2634112

[51] Müller K, Job C, Belghazi M, Job D, Leubner-Metzger G (2010) Proteomics reveal tissue-specific features of the cress (Lepidium sativum L.) endosperm cap proteome and its hormone-induced changes during seed germination. Proteomics 10(3): 406–416. 19943265 10.1002/pmic.200900548

[52] Chaudhuri A, Singh KL, Kar RK (2013) Interaction of Hormones with Reactive Oxygen Species in Regulating Seed Germination of Vigna radiata (L.) Wilczek. J Plant Biochem Physiol 1: 1–5.

[53] Müller K, Carstens AC, Linkies A, Torres MA, Leubner-Metzger G (2009) The NADPH-oxidase AtrbohB plays a role in Arabidopsis seed after-ripening”. New Phytol 184(4): 885–897. 19761445 10.1111/j.1469-8137.2009.03005.x

