# Supplementary figures and images for: OsRACK1 Is Involved in Abscisic Acid- and H2O2-Mediated Signaling to Regulate Seed Germination in Rice (Oryza sativa, L.)
Source: PLoS One. 2014 May 27;9(5):e97120. doi: 10.1371/journal.pone.0097120 (PMC4035261; doi:10.1371/journal.pone.0097120)

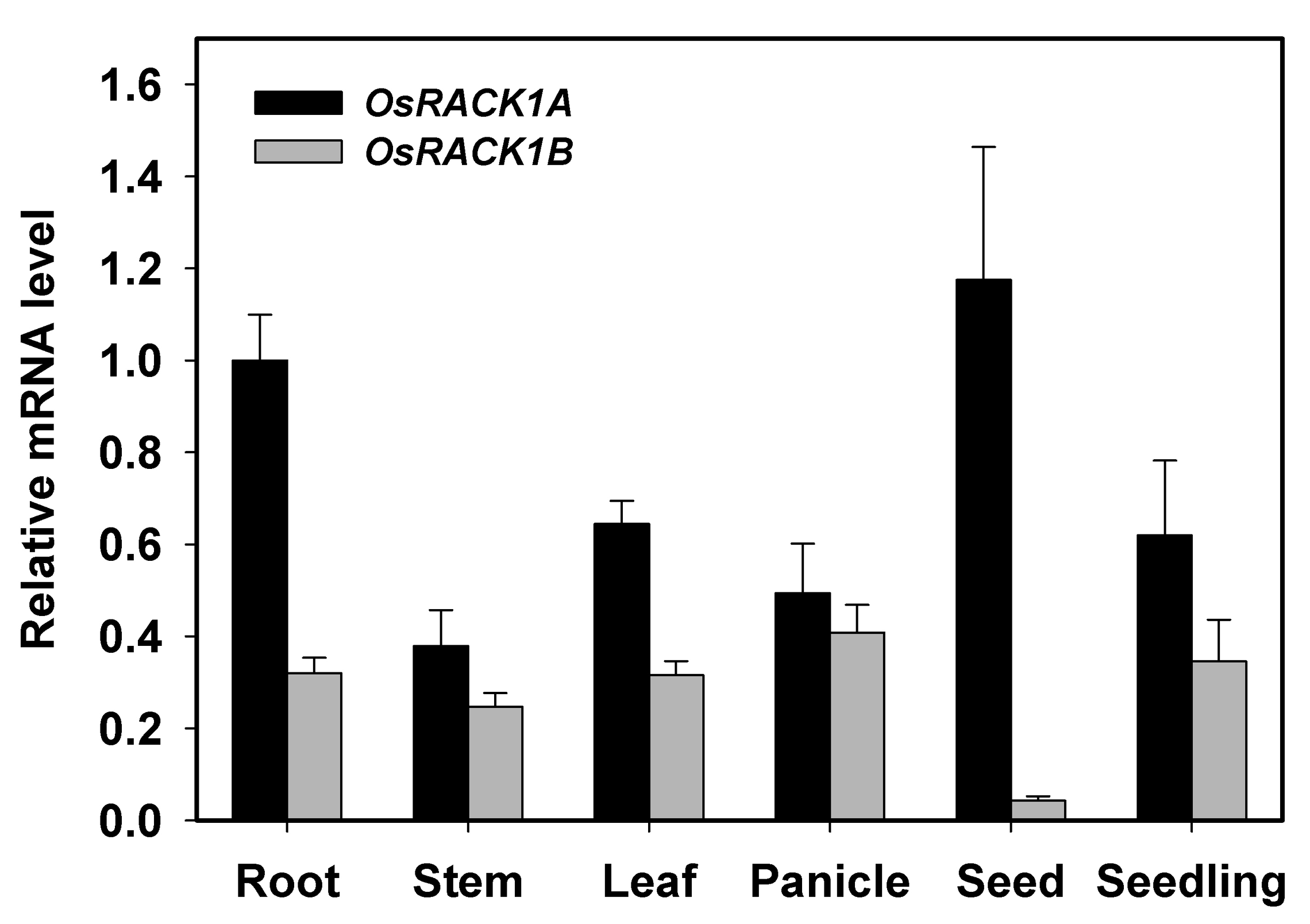

Supplement: Figure S1 — Quantitative RT-PCR analysis of OsRACK1A and OsRACK1B expression in rice tissues. Total RNA isolated from root, stem, leaf, panicle, dry seed or seedling (2 weeks) was used as template for qRT-PCR. Relative expression levels were calculated and normalized with respect to OsActin7 (LOC_Os11g06390). Results shown are means of three biological replications±SE. (TIF) [file pone.0097120.s001.tif]

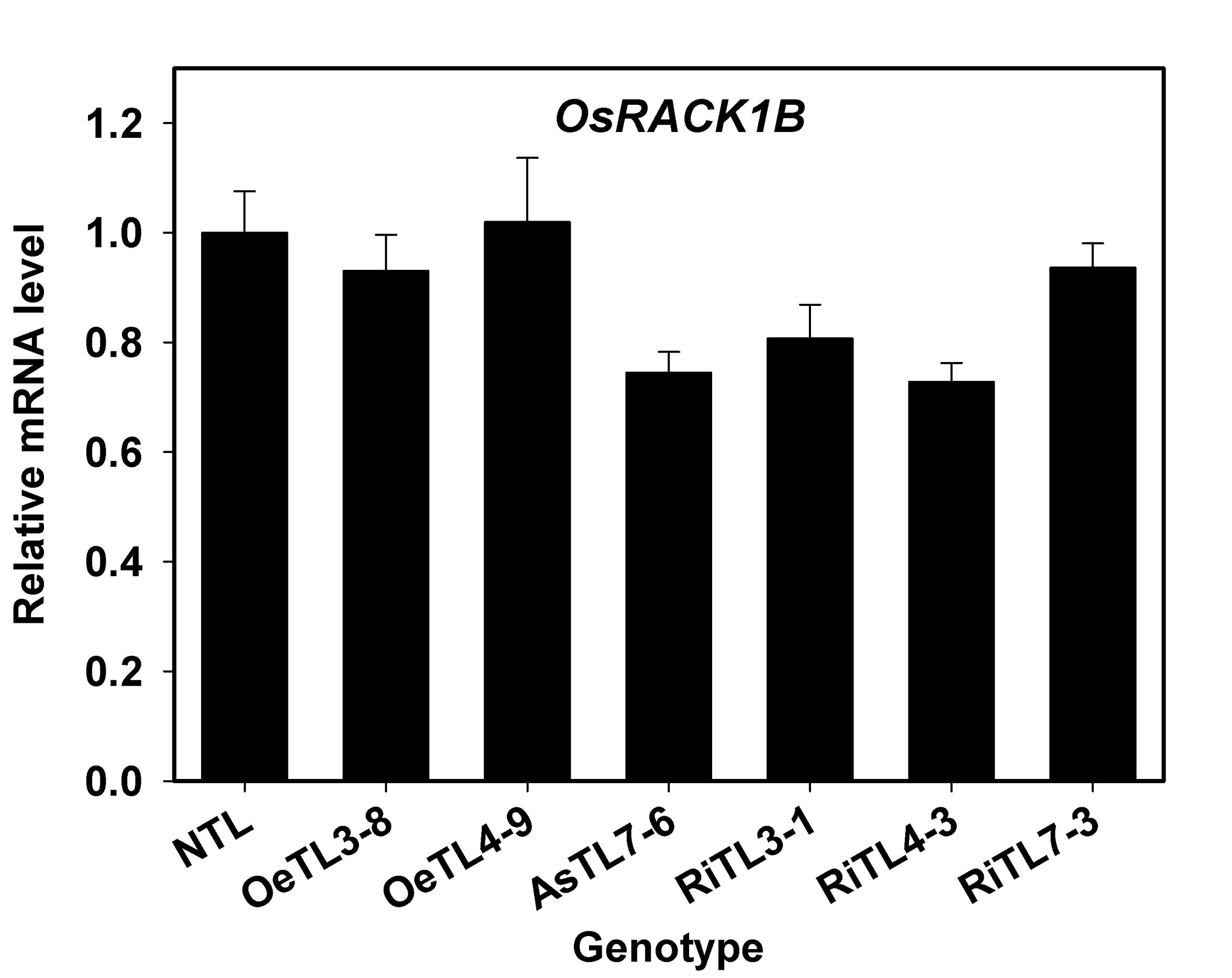

Supplement: Figure S2 — Expressional profile of OsRACK1B in selected transgenic rice lines. OsRACK1B expression was monitored in seedlings of wildtype (non-transgenic lines, NTL), OsRACK1A over-expressed transgenic lines (OeTL), anti-sense transgenic lines (AsTL) and RNA-interfered transgenic lines (RiTLs), respectively. Relative expression levels were calculated and normalized with respect to OsActin7 (LOC_Os11g06390). Results shown are means of three biological replications±SE. (TIF) [file pone.0097120.s002.tif]

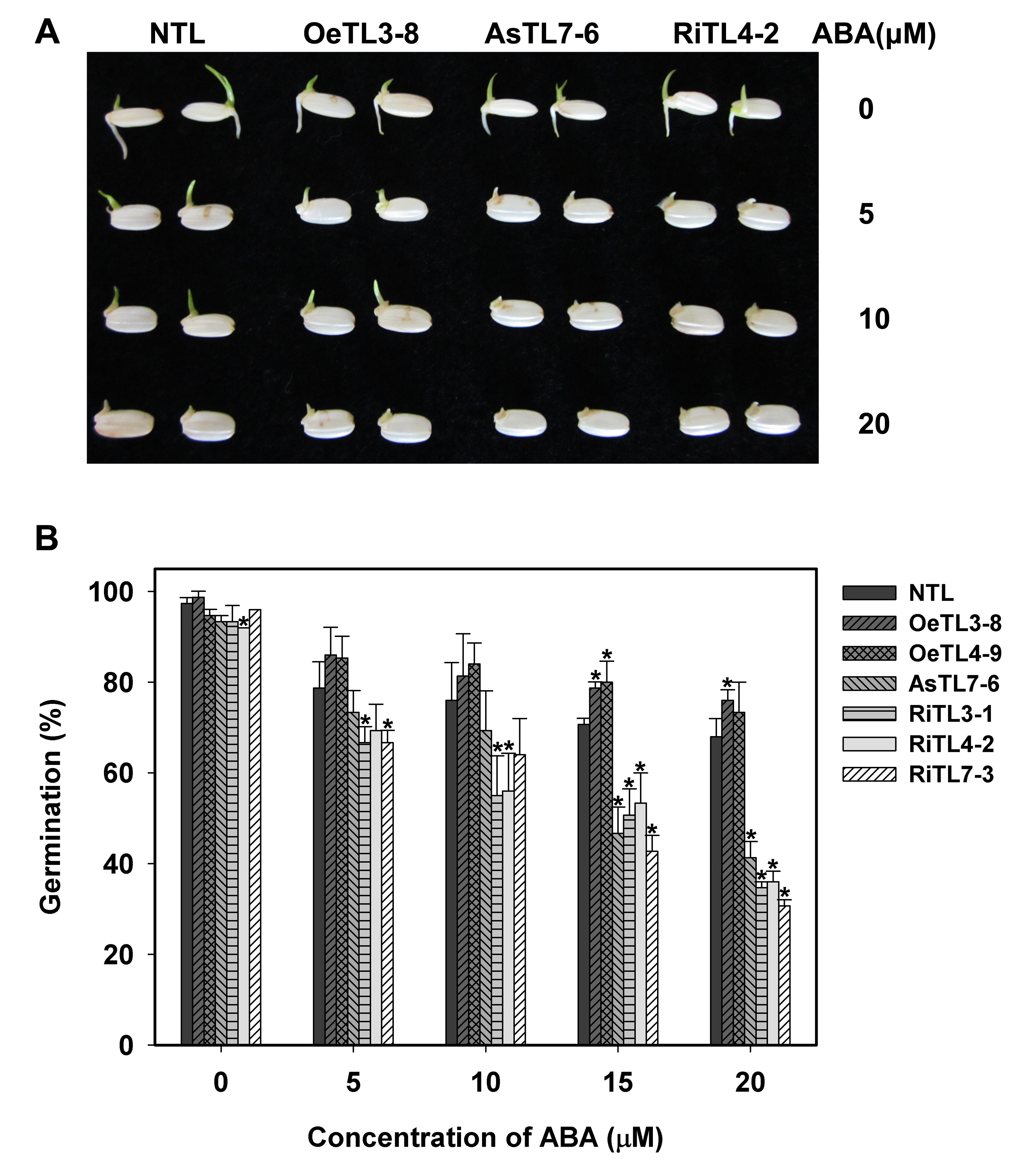

Supplement: Figure S3 — Effects of different concentration of ABA treatments on seed germination of different transgenic lines. A. Morphology of germinating seeds in the different concentration of ABA (0, 5, 10 and 20 µM) treatment for 24 hours. B. Seed germination rates under 0, 5, 10 and 3 µM ABA treatment for 24 hours. Each value is the mean ± standard error of at least 50 seeds. Asterisks (*) indicate significant difference (P<0.05) between the seed germination of transgenic lines compared with the wildtype. (TIF) [file pone.0097120.s003.tif]

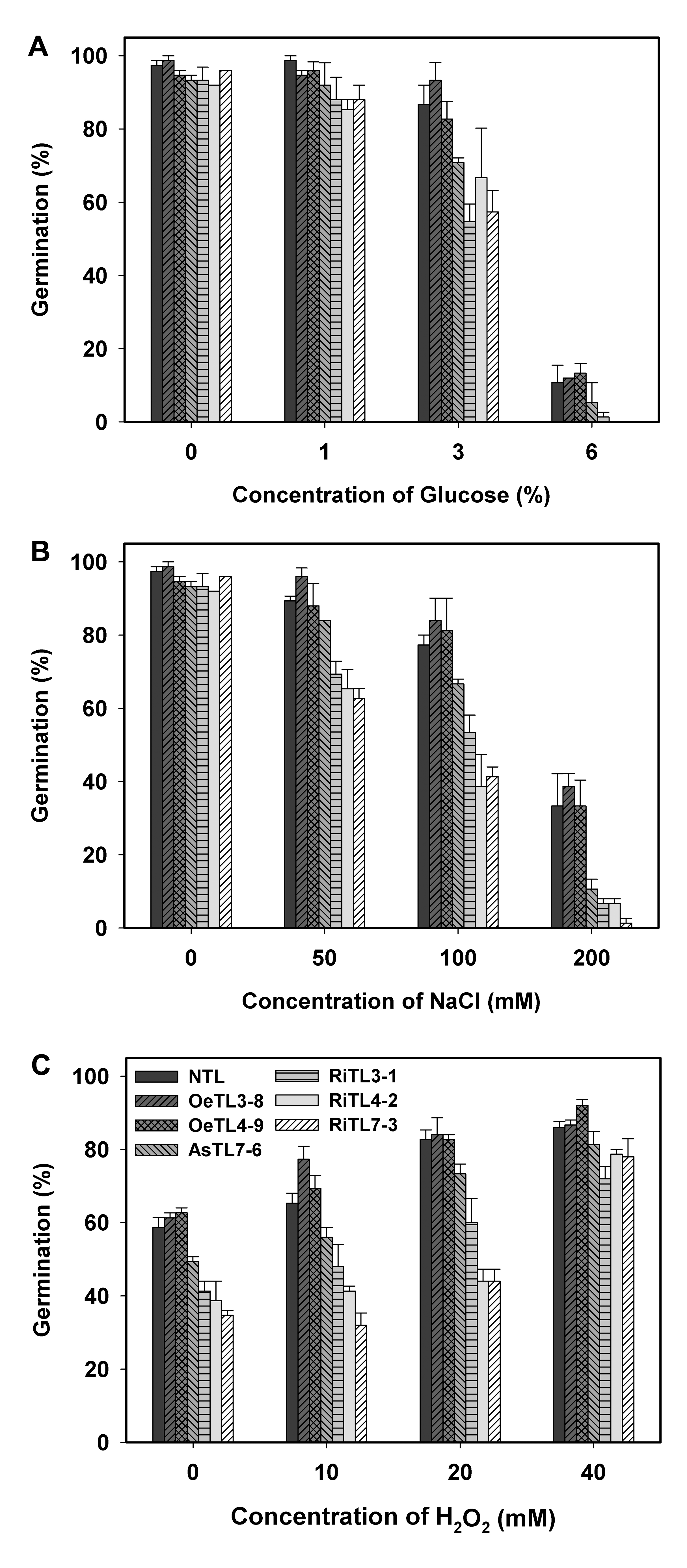

Supplement: Figure S4 — Effects of different concentrations of glucose, NaCl and H2O2 on seed germination of different transgenic lines. Sterilized seeds were germinated at 28°C on sterile filter papers in the petri dishes containing different concentrations of glucose (0, 1, 3 and 6 mM), NaCl (0, 50, 100 and 200 mM) or H2O2 (0, 10, 20 and 40 mM). Germination (based on radicals >2 mm) was recorded at the indicated time points. Fifty seeds per genotype were used. Data shown are means of three biological replications±SE. Asterisks (*) indicate significant difference (P<0.05) between the seed germination of transgenic lines compared with the wildtype. (TIF) [file pone.0097120.s004.tif]

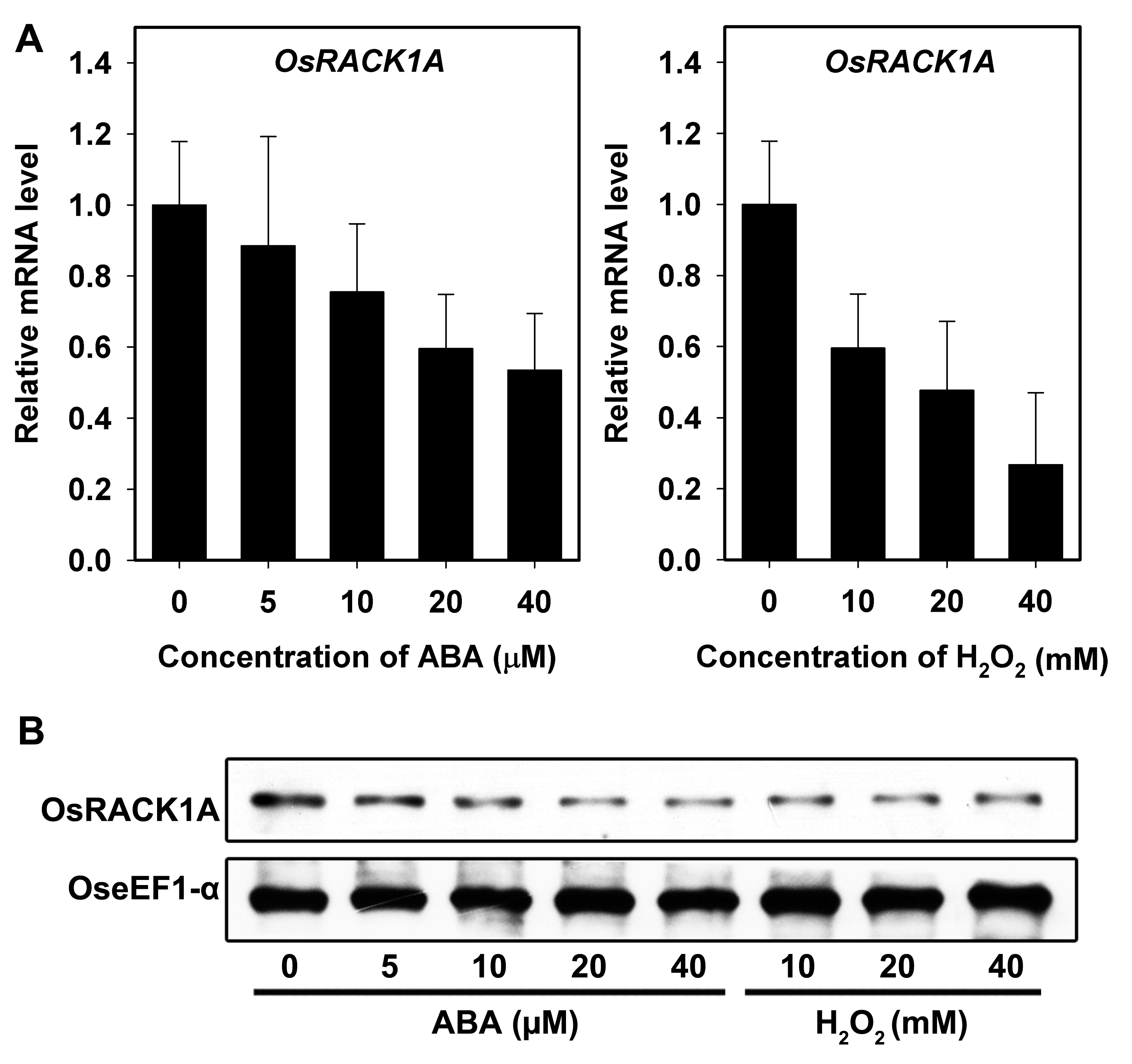

Supplement: Figure S5 — Effects of ABA and H2O2 on expressional profile of OsRACK1A genes (A) and proteins (B) in selected transgenic rice lines. Two-week-old seedlings were treatment with different concentration of ABA (5, 10, 20 and 40 µM) or H2O2 (10, 20 and 40 mM) for 24 h before RNA or protein extraction. A. Relative gene expression levels were calculated and normalized with respect to OsActin7 (LOC_Os11g06390). B. OsRACK1A expression was analyzed by incubating isolated proteins with polyclonal antibodies against OsRACK1A or OseEF1-α (as reference). (TIF) [file pone.0097120.s005.tif]
