# Supplementary material for: OsRACK1 Is Involved in Abscisic Acid- and H2O2-Mediated Signaling to Regulate Seed Germination in Rice (Oryza sativa, L.)
Source: PLoS One. 2014 May 27;9(5):e97120. doi: 10.1371/journal.pone.0097120 (PMC4035261; doi:10.1371/journal.pone.0097120)
Supplement: Table S1 — Gene specific primers used in quantitative real-time PCR (qRT-PCR). (DOCX) [file pone.0097120.s006.docx]

**Table S1. Gene specific primers used in quantitative real-time PCR (qRT-PCR)**

| Gene | GenBank accession No. | Primers |
| --- | --- | --- |
| *OsActin7* | NM_001072362 | F: ACAGGTATTGTGTTGGACTCTGG  R: AGTAACCACGCTCCGTCAGG |
| *OsRACK1A* | NM_001050445 | F: AGGGATCTGTTTTGCACCAT  R: GAGAGAAGCACCATGGATCG |
| *OsRACK1B* | NM_001062789 | F: TCTTCGCTGGCTACACTG  R: AATTCACCACCTAGATTGC |
| *Ramy1A* | AK101744 | F: TCCCTTTCGGTCCTCATCG  R: ACCCGCCATTCTCCTTCC |
| *Ramy3D* | M24287 | F: CGGGATAGTCATGCTCAAAC  R: ACGCTACAATCGGATACAAAA |
| *Rboh2*  *Rboh5*  *Rboh9* | AK103747  AK120905  NM_001073486 | F: CAATGTCCAGCAGTGTCT  R: GAGTTCTCGTGTCCAATCA  F: TGGAGAGTGGCTATACCTTA  R: CAACAGACGGACAGAATAAC  F: TCAACTTCCACAAGGTAACA  R: CGCTTCTACTGGTGATAATAC |
